# Spermatorrhœa and Hypochondriasis

**Published:** 1882-12

**Authors:** 


### SPERMATORRHŒA AND HYPOCHONDRIASIS.

Dr. S. V. Cobb, of Ashland, O., reports two cases which he has treated with Horsford's Acid Phosphate with marked success. The first—Spermatorrhœa, accompanied with hypochondriasis and dyspeptic tendency. The other case in which he used it was in connection with wine as a tonic, in the convalescence of a lady eighty years of age from a severe attack of dysenteria. The nervous symptoms seemed to be controlled well by it.

